# Heparin-induced thrombocytopenia: an update

**DOI:** 10.1186/1477-9560-3-14

**Published:** 2005-10-04

**Authors:** Massimo Franchini

**Affiliations:** 1 Servizio di Immunoematologia e Trasfusione, Azienda Ospedaliera di Verona, Verona, Italy

**Keywords:** Platelets, heparin, thrombosis

## Abstract

Heparin-induced thrombocytopenia (HIT) is the most important and most frequent drug-induced, immune-mediated type of thrombocytopenia. It is associated with significant morbidity and mortality if unrecognized. In this review, we briefly discuss the main features of heparin-induced thrombocytopenia, particularly analyzing the most recent advances in the pathophysiology, diagnosis and treatment of this syndrome.

## Introduction

Heparin is a drug widely used for thromboprophylaxis or treatment in many clinical situations, including cardiovascular surgery and invasive procedures, acute coronary syndromes, venous thromboembolism, atrial fibrillation, peripheral occlusive disease, dialysis and during extracorporeal circulation [1,2]. However, it can cause serious adverse effects, including heparin-induced thrombocytopenia (HIT) which is a common, serious and potentially life-threatening condition [3-6]. Unfortunately, because thrombocytopenia is common in hospitalized patients and can be caused by a variety of factors [7], HIT often remains unrecognized.

Heparin-induced thrombocytopenia is defined as a decrease in platelet count during or shortly following exposure to heparin [8]. Two different types of HIT are recognized. The first, HIT type I (also called heparin-associated thrombocytopenia in the past), is a benign form not associated with an increased risk of thrombosis. The mechanism of HIT type I is still unknown but it is likely to be non-immune, probably related to its platelet pro-aggregating effect. This form of HIT affects up to 10% of patients under treatment with heparin and is characterized by a mild and transient asymptomatic thrombocytopenia (rarely less than 100,000 platelets/μL) that develops early (usually within the first two days of starting heparin) and disappears equally quickly once the heparin is withdrawn. The second form of HIT, HIT type II, is immune-mediated and associated with a risk of thrombosis. It has recently been proposed that the term "HIT type I" be changed to "non-immune heparin associated thrombocytopenia" and that the term "HIT type II" be changed to "HIT" to avoid confusion between the two syndromes [9].

In this review we briefly analyze the main characteristics of the clinically relevant, immune-mediated, second type of HIT, focusing particularly on the epidemiology, pathophysiology, clinical manifestations and treatment of this syndrome. For simplicity and also in accordance with the new recommendations, in the following the term HIT refers to HIT type II.

## Incidence

Heparin-induced thrombocytopenia is the most important of the immune-mediated, drug-induced thrombocytopenias. Recent data show that up to 8% of heparinized patients will develop the antibody associated with HIT [10] and that approximately 1–5% of patients on heparin will progress to develop HIT with thrombocytopenia [11,12], suffering from venous and/or arterial thrombosis in at least one-third of cases [13,14]. In a recent analysis of 598 consecutive hospitalized medical patients treated with subcutaneous unfractionated heparin, Girolami and colleagues diagnosed five cases of HIT (0.8%) [15]. In general, the antibodies occur more frequently in patients undergoing cardiovascular surgery than those undergoing orthopedic surgery, and in post-surgical patients than in medical patients. HIT antibodies are also more frequent in patients receiving unfractionated heparin (UFH) than in those treated with low molecular weight heparin (LMWH) [16,17], although it must be highlighted that antibodies developing in patients receiving UFH frequently cross-react with LMWH [13]. In a study conducted on 665 patients undergoing elective hip arthroplasty who had been randomized to receive either UFH or LMWH for thromboprophylaxis, Warkenitin and colleagues reported that HIT occurred in 9 of 332 patients who received UFH and in none of 333 patients who received LMWH (2.7% versus 0%, P = 0.0018) [10]. In addition, development of heparin-dependent antibodies and thrombotic events associated with thrombocytopenia were more common in patients treated with UFH than in those treated with LMWH.

## Pathophysiology

**Figure 1:**
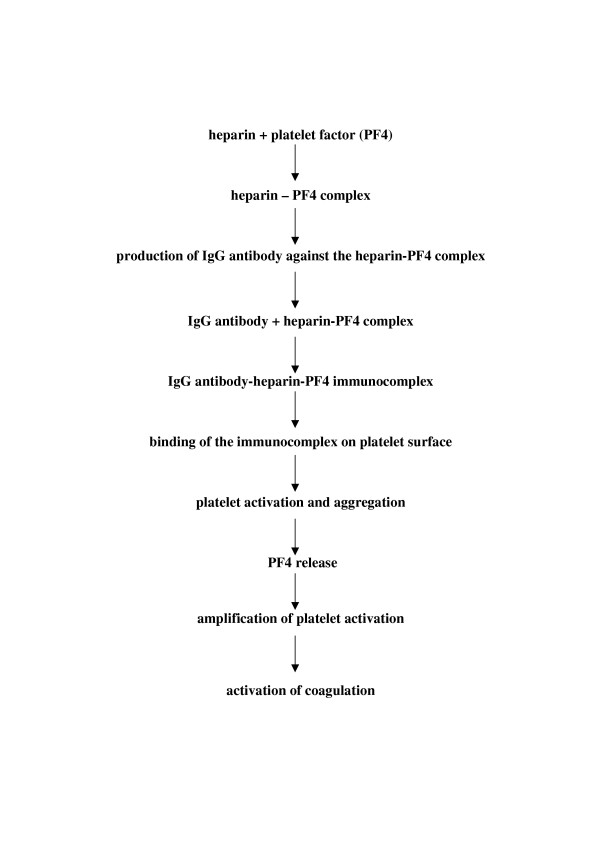
Pathophysiology of heparin induced thrombocytopenia.

The mechanism underlying heparin-induced thrombocytopenia is an immune response [18,19]. The principal antigen is a complex of heparin and platelet factor 4 (PF4). Platelet factor 4 is a small positively charged molecule of uncertain biological function normally found in α-granules of platelets. When platelets are activated, PF4 is released into the circulation and some of it binds to the platelet surface. Because of opposite charges, heparin and other glycosaminoglycans bind to the PF4 molecules, exposing neoepitopes that act as immunogens leading to antibody production. In fact, patients who develop HIT produce an IgG antibody against the heparin-PF4 complex, which binds to the complex on platelet surface through the Fab region [20]. The Fc portion of the HIT antibody can then bind to the platelet Fc receptor and this interaction triggers activation and aggregation of the platelets. Activated platelets release PF4, thus perpetuating the cycle of heparin-induced platelet activation. In addition, the platelet activation leads to the production of prothrombotic platelet microparticles which promote coagulation. Finally, as a result of the presence of heparin-like molecules (heparan sulfate) on the surface of endothelial cells, the HIT antibody-PF4-heparan sulfate complexes formed on the endothelial surface may induce tissue factor expression with further activation of the coagulation cascade and thrombin generation [21,22]. Thrombocytopenia in HIT is largely due to the clearance of activated platelets and antibody-coated platelets by the reticulo-endothelial system [1]. Figure 1 illustrates the pathophysiology of HIT.

## Clinical features and diagnosis

The onset of heparin-induced thrombocytopenia may be rapid or delayed. The platelet count in patients with pre-existing heparin-PF4 antibodies from a previous exposure and sensitization to heparin may decrease within the first 3 days or even hours after re-exposure to heparin (rapid-onset HIT) [23]. However, in patients receiving heparin for the first time, the onset of thrombocytopenia usually occurs 5 to 10 days after the administration of the heparin. Conversely, in delayed-onset HIT, the thrombocytopenia occurs 5 or more days after heparin withdrawal [24].

The thrombocytopenia in HIT is usually moderate in severity, with a median platelet count being between 50 and 80 × 10^9^/L, although the nadir platelet count can remain at a level considered normal (i.e. > 150 × 10^9^/L) but having dropped by 50% or more with respect to the pre-heparin value. The platelet count starts to rise 2 to 3 days after discontinuing heparin and usually returns to normal within 4 to 10 days. The antibody disappears within 2 to 3 months after cessation of heparin therapy [11]. Although HIT does not invariably recur during subsequent re-exposure to heparin, future use of heparin is contraindicated [25]. Despite thrombocytopenia, bleeding is rare [2]. Contrariwise, HIT is strongly associated with thrombosis, which frequently leads to the recognition of HIT [26]. Thrombosis in HIT is associated with a mortality of approximately 20–30%, with an equal percentage of patients becoming permanently disabled by amputation, stroke or other causes [27]. Thromboembolic complications can be venous, arterial, or both and include deep venous thrombosis, pulmonary embolism, myocardial infarction, thrombotic stroke and occlusion of limb arteries [28]. However, the type and site of thrombosis depends on the patient's clinical profile. For example, deep vein thrombosis and pulmonary embolism occur very frequently in postoperative patients who are already prone to developing venous thromboembolism [10]. In fact, Warkentin and colleagues reported that the incidence of deep vein thrombosis in orthopedic patients who received heparin for thromboprophylaxis was 17.8%, but that this incidence increased dramatically to 88.9% among patients who developed HIT [10]. Similarly, patients with central venous catheters and HIT develop upper limb venous thrombosis more frequently than those without HIT [1]. In some cases thrombosis of the cerebral venous sinuses can occur, giving rise to a clinical picture of severe headache and progressive neurological deficits [29]. In contrast, arterial thrombosis occurs more frequently than venous thrombosis in HIT patients receiving heparin for cardiovascular diseases [30]. Furthermore, areas of necrosis developing at the site of heparin injections can be a manifestation of HIT, and are not necessarily associated with thrombocytopenia [31]. Platelet activation and thrombosis due to heparin-dependent, platelet-activating IgG have been shown to be the underlying pathogenic mechanisms of this complication [31]. In some cases, thrombosis may be generalized leading to a syndrome resembling disseminated intravascular coagulation [32].

Finally, in some patients with HIT resistance to heparin may occur, meaning that an increasing dose of heparin dose is required to maintain the activated partial thromboplastin time (aPTT) within the therapeutic range [13].

The diagnosis of HIT remains a clinical one, supported by confirmatory laboratory testing [5,6]. The criteria include: a) thrombocytopenia (i.e., a drop of the platelet count to below 100 × 10^9^/L or a drop of > 50% from the patient's baseline platelet count); b) the exclusion of other causes of thrombocytopenia; c) the resolution of thrombocytopenia after cessation of heparin [1]. As regards the laboratory tests, HIT-antibodies can be demonstrated *in vitro *by functional tests and immunoassays [4,8]. Functional tests, which measure platelet activity in the presence of the patient's serum and heparin, include heparin-induced platelet aggregation (HIPA) and the serotonin release assay (SRA). Although the HIPA test is easier to perform and thus more commonly used, the SRA is more sensitive, albeit more complex, technically demanding and not readily available in most centers, and is therefore considered the "gold standard" [1]. The immunoassays utilize immunoenzymatic tests (enzyme-linked immunesorbent assay, ELISA) to detect the HIT antibody that binds to the PF4/heparin complex. Immunoassays are technically easier to perform than the functional assays and are also more sensitive [1]. On the other hand, comparative and prospective studies have demonstrated that functional tests are more specific than enzyme immunoassays and thus, being better at detecting the clinically significant HIT antibodies, are more helpful in the diagnosis of HIT [3].

## Treatment

When HIT is suspected clinically, immediate cessation of all formulations of heparin is mandatory, but this will neither stop continuing thrombin generation nor avoid subsequent thrombotic events, which occur in as many as 40–50% of the patients over the next several days or weeks [33]. Interestingly, in a retrospective analysis of 113 patients with HIT, Wallis and colleagues [34] found that early heparin cessation (0.7 ± 0.6 days) was no more effective in reducing morbidity and mortality than was late heparin cessation (5 ± 3 days), thus indicating that heparin cessation alone is not sufficient treatment for HIT. In fact, the appropriate treatment for HIT requires immediate removal of the trigger (heparin cessation) as well as control of the thrombin storm of HIT (by providing appropriate alternative anticoagulation). Currently, three non-heparin anticoagulants that do not cross-react with HIT antibodies, danaparoid, lepirudin and argatroban, are available for alternative anticoagulation in HIT [35-41]. These drugs are immediately active and either inhibit thrombin directly or inhibit thrombin generation. As reported above, LMWH cannot be used in patients with HIT because of the strong cross-reactivity of the HIT antibody with the LMW heparin/PF4 complex. The duration of treatment for patients with HIT is not well defined. However, anticoagulation treatment is required for at least 2 to 3 months to prevent recurrence of thrombosis. Oral anticoagulation with warfarin should be initiated until substantial platelet count recovery has occurred and while the patient is receiving danaparoid or a thrombin-specific inhibitor (an overlap of at least 5 days is recommended) [33]. In fact, it has recently become known that HIT patients who are switched to warfarin alone after the discontinuation of heparin may paradoxically have worsening thrombosis and develop venous limb gangrene and skin necrosis [42]. The mechanism appears to be a warfarin-induced marked decrease in protein C before prothrombin levels are adequately suppressed [2].

Danaparoid has been successfully used as a replacement for heparin in patients with HIT [43]. This anticoagulant is composed of a mixture of three glycosaminoglycans (heparin sulfate, dermatan sulfate and chondroitin sulfate) and, via antithrombin, inhibits anti-FXa activity. In a prospective randomized study conducted by Chong and colleagues [41], danaparoid was shown to be more effective than dextran 70 in the treatment of HIT-associated venous and arterial thrombosis. In a compassionate use program, more than 460 patients with HIT-associated thrombosis were treated with danaparoid with a success rate of over 90% [44]. For treatment of HIT, danaparoid is given as an intravenous bolus dose of 2500 U followed by 400 U/hour for 4 hours, then 300 U/hour for 4 hours and subsequently 200 U/hour until anticoagulation is no longer required, adjusting the dose to maintain plasma anti-Xa levels within 0.5–0.8 U/mL. Alternatively, danaparoid can be administered subcutaneously using a bolus of 1250 U followed by 2000 U twice a day [1].

Recombinant hirudin (lepirudin), an anticoagulant protein originally produced by the medicinal leech, inhibits thrombin directly [1]. In a meta-analysis of three prospective multicenter trials including 91 patients with laboratory-confirmed acute HIT treated with lepirudin, Lubenow and colleagues [45] found that the incidence of the combined end-point of death, new thromboembolic complications and limb amputation was significantly lower in the lepirudin-treated patients than in a contemporaneous control group not treated with lepirudin. Currently recommended doses are 0.4 mg/kg as a bolus followed by 0.15 mg/kg/hour adjusting the dose to achieve an aPTT of 1.5 to 3 times the baseline value [33]. In a retrospective study of 175 lepirudin-treated HIT patients and 126 danaparoid-treated HIT patients, Farner and colleagues [46] found no significant difference in the same combined end-points between the two groups.

Argatroban, an arginine-based synthetic anticoagulant, is a direct inhibitor of thrombin that reversibly binds the catalytic site of thrombin [13,47]. A multicenter, prospective study conducted on 304 HIT patients receiving argatroban found that the above mentioned combined end-points were significantly reduced in argatroban-treated patients compared to in historical controls [40]. The recommended initial dose is 2 μg/kg/minute given intravenously and adjusted to achieve an aPTT 1.5 to 3 times the baseline value. Since argatroban is cleared by the liver, lepirudin, which is cleared through the kidneys, should be preferred in patients with liver disease. Vice versa, argatroban would be a better initial choice in patients with renal insufficiency. Thrombin-specific inhibitors also prolong the INR, but this effect is particularly pronounced with argatroban [48]. Thus, during the transition from argatroban to oral anticoagulation special precautions must be taken [49,50].

Finally, there is recent evidence that a novel synthetic heparin pentasaccharide, fondaparinux, which does not cross-react with HIT antibodies [51], can be successfully used for the treatment of patients with HIT [52,53]. However, additional controlled clinical studies are required to further evaluate the safety and efficacy of this agent in patients with HIT.

## Conclusion

The analysis of the literature data reveals that heparin-induced thrombocytopenia is not only a common but also a serious complication of heparin therapy with a high rate of morbidity and mortality. Its prompt clinical and laboratory recognition is thus essential in order to stop heparin use immediately and commence an alternative anticoagulant. The low molecular weight heparinoid, danaparoid, and the thrombin-specific inhibitors, lepirudin and argatroban, have been shown to be effective in HIT patients.

## References

[B1] Chong BH (2003). Heparin-induced thrombocytopenia. J Thromb Haemost.

[B2] Jang I-K, Hursting HJ (2005). When heparins promote thrombosis. Review of heparin-induced thrombocytopenia. Circulation.

[B3] Warkentin TE (2003). Heparin-induced thrombocytopenia: pathogenesis and management. Br J Haematol.

[B4] Warkentin TE (2004). Heparin-induced thrombocytopenia. Diagnosis and management. Circulation.

[B5] Cines DB, Bussel JB, McMillan RB, Zehnder JL (2004). Congenital and acquired thrombocytopenia. Hematology (Am Soc Hematol Educ Program).

[B6] Warkentin TE, Greinacher A (2004). Heparin-induced thrombocytopenia: recognition, treatment, and prevention: the Seventh ACCP Conference on Antithrombotic and Thrombolytic Therapy. Chest.

[B7] Strauss R, Wehler M, Mehler K, Kreutzer D, Koebnick C, Hahn EG (2002). Thrombocytopenia in patients in the medical intensive care unit: bleeding prevalence, transfusion requirements, and outcome. Crit Care Med.

[B8] Warkentin TE (2004). An overview of the heparin-induced thrombocytopenia syndrome. Semin Thromb Hemost.

[B9] Rice L (2004). Heparin-induced thrombocytopenia: myths and misconceptions (that will cause trouble for you and your patient). Arch Intern Med.

[B10] Warkentin TE, Levine MN, Hirsh J (1995). Heparin-induced thrombocytopenia in patients treated with low-molecular-weight heparin or unfractionated heparin. N Engl J Med.

[B11] Kelton JG (2002). Heparin-induced thrombocytopenia: an overview. Blood Rev.

[B12] Baglin TP (1997). Heparin-induced thrombocytopenia/thrombosis syndrome (HIT): diagnosis and treatment. Platelets.

[B13] Comunale ME, van Cott EM (2004). Heparin-induced thrombocytopenia. Int Anesthesiol Clin.

[B14] Nand S, Wong W, Yuen B, Yetter A, Schmulbach E, Gross Fisher S (1997). Heparin-induced thrombocytopenia with thrombosis: incidence, analysis of risk factors, and clinical outcomes in 108 consecutive patients treated at a single institution. Am J Hematol.

[B15] Girolami B, Prandoni P, Stefani PM (2003). The incidence of heparin-induced thrombocytopenia in hospitalized medical patients treated with subcutaneous unfractionated heparin: a prospective cohort study. Blood.

[B16] Lindhoff-Last E, Eichler P, Stein M (2000). A prospective study on the incidence and clinical relevance of heparin-induced antibodies in patients after vascular surgery. Thromb Res.

[B17] Locke CFS, Dooley J, Gerber J (2005). Rates of clinically apparent heparin-induced thrombocytopenia for unfractionated heparin vs. low molecular weight heparin in non-surgical patients are low and similar. Thrombosis J.

[B18] Reilly RF (2003). The pathophysiology of immune-mediated heparin-induced thrombocytopenia. Semin Dial.

[B19] Arepally G, Cines DB (2002). Pathogenesis of heparin-induced thrombocytopenia and thrombosis. Autoimmun Rev.

[B20] Kelton JG, Smith JW, Warkentin TE, Hayward CP, Denomme GA, Horsewood P (1994). Immunoglobulin G from patients with heparin-induced thrombocytopenia binds to a complex of heparin and platelet factor 4. Blood.

[B21] Cines DB, Tomaski A, Tannenbaum S (1987). Immune endothelial-cell injury in heparin-associated thrombocytopenia. N Engl J Med.

[B22] Visentin GP, Ford SE, Scott JP, Aster RH (1994). Antibodies from patients with heparin-induced thrombocytopenia/thrombosis are specific for platelet factor 4 complexed with heparin or bound to endothelial cells. J Clin Invest.

[B23] Warkentin TE, Kelton JG (2001). Temporal aspects of heparin-induced thrombocytopenia. N Engl J Med.

[B24] Warkentin TE, Kelton JG (2001). Delayed-onset heparin-induced thrombocytopenia and thrombosis. Ann Intern Med.

[B25] Bell WR (1988). Heparin-associated thrombocytopenia and thrombosis. J Lab Clin Med.

[B26] Warkentin TE, Kelton JG (1996). A 14-year study of heparin-induced thrombocytopenia. Am J Med.

[B27] Greinacher A (1995). Antigen generation in heparin-associated thrombocytopenia: the nonimmunologic type and the immunologic type are closely linked in their pathogenesis. Semin Thromb Hemost.

[B28] Warkentin TE (1999). Heparin-induced thrombocytopenia: a clinicopathologic syndrome. Thromb Haemost.

[B29] Meyer-Lindenberg A, Quenzel EM, Bierhoff E, Wolff H, Schindler E, Biniek R (1997). Fatal cerebral venous sinus thrombosis in heparin-induced thrombotic thrombocytopenia. Eur Neurol.

[B30] Boshkov LK, Warkentin TE, Hayward CP, Andrew M, Kelton JG (1993). Heparin-induced thrombocytopenia and thrombosis: clinical and laboratory studies. Br J Haematol.

[B31] Warkentin TE (1996). Heparin-induced skin lesions. Br J Haematol.

[B32] Klein HG, Bell WR (1974). Disseminated intravascular coagulation during heparin therapy. Ann Intern Med.

[B33] Alving BM (2003). How I treat heparin-induced thrombocytopenia and thrombosis. Blood.

[B34] Wallis DE, Workman DL, Lewis BE, Steen L, Pifarre R, Moran JF (1999). Failure of early heparin cessation as treatment for heparin-induced thrombocytopenia. Am J Med.

[B35] Chong BH (1995). Diagnosis, treatment and pathophysiology of immune-mediated thrombocytopenia. Crit Rev Oncol Hematol.

[B36] Magnani HN (1993). Heparin-induced thrombocytopenia (HIT): an overview of 230 patients treated with orgaran (Org 10172). Thromb Haemost.

[B37] Greinacher A, Janssens U, Berg G (1999). Lepirudin (recombinant hirudin) for parenteral anticoagulation in patients with heparin-induced thrombocytopenia. Heparin-Associated Thrombocytopenia Study (HAT) investigators. Circulation.

[B38] Greinacher A, Volpel H, Janssens U (1999). Recombinant hirudin (lepirudin) provides safe and effective anticoagulation in patients with heparin-induced thrombocytopenia: a prospective study. Circulation.

[B39] Greinacher A, Eichler P, Lubenow N, Kwasny H, Luz M (2000). Heparin-induced thrombocytopenia with thromboembolic complications: meta-analysis of 2 prospective trials to assess the value of parenteral treatment with lepirudin and its therapeutic aPTT range. Blood.

[B40] Lewis BE, Wallis DE, Berkowitz SD, for the ARG-911 Study Investigators (2001). Argatroban anticoagulant therapy in patients with heparin-induced thrombocytopenia. Circulation.

[B41] Chong BH, Gallus AS, Cade JF, for the Australian HIT Study Group (2001). Prospective randomised open-label comparison of danaparoid with dextran 70 in the treatment of heparin-induced thrombocytopaenia with thrombosis: a clinical outcome study. Thromb Haemost.

[B42] Warkentin TE, Elavathil LJ, Hayward CP, Johnston MA, Russett JI, Kelton JG (1997). The pathogenesis of venous limb gangrene associated with heparin-induced thrombocytopenia. Ann Intern Med.

[B43] Ortel TL, Gockerman JP, Califf RM (1992). Parenteral anticoagulation with the heparinoid Lomoparan (Org 10172) in patients with heparin induced thrombocytopenia and thrombosis. Thromb Haemost.

[B44] Ortel TL, Chong BH (1998). New treatment options for heparin-induced thrombocytopenia. Sem Hematol.

[B45] Lubenow N, Eichler P, Lietz T, Farner B, Greinacher A (2004). Lepirudin for prophylaxis of thrombosis in patients with acute isolated heparin-induced thrombocytopenia: an analysis of 3 prospective studies. Blood.

[B46] Farner B, Eichler P, Kroll H, Greinacher A (2001). A comparison of danaparoid and lepirudin in heparin-induced thrombocytopenia. Thromb Haemost.

[B47] Hirsh J, Heddle N, Kelton JG (2004). Treatment of heparin-induced thrombocytopenia: a critical review. Arch Intern Med.

[B48] Gosselin RC, Dager WE, King JH (2004). Effect of direct thrombin inhibitors, bivalirudin, lepirudin, and argatroban, on prothrombin time and INR values. Am J Clin Pathol.

[B49] Sheth SB, DiCicco RA, Hursting MJ, Montague T, Jorkasky DK (2001). Interpreting the international normalized ratio (INR) in individuals receiving argatroban and warfarin. Thromb Haemost.

[B50] Hursting MJ, Lewis BE, Macfarlane DE (2005). Transitioning from argatroban to warfarin therapy in patients with heparin-induced thrombocytopenia. Clin Appl Thromb Hemost.

[B51] Savi P, Chong BH, Greinacher A (2005). Effect of fondaparinux on platelet activation in the presence of heparin-dependent antibodies: a blinded comparative multicenter study with unfractioned heparin. Blood.

[B52] Harenberg J, Jorg I, Fenyvesi T (2005). Treatment of heparin-induced thrombocytopenia with fondaparinux. Haematologica.

[B53] Kuo KHM, Kovacs MJ (2005). Successful treatment of heparin induced thrombocytopenia (HIT) with fondaparinux. Thromb Haemost.

